# Intermittent Fasting After Spinal Cord Injury Does Not Improve the Recovery of Baroreflex Regulation in the Rat

**DOI:** 10.3389/fphys.2020.00865

**Published:** 2020-07-22

**Authors:** Matthew R. Zahner, Eric Beaumont

**Affiliations:** ^1^Department of Health Sciences, College of Public Health, East Tennessee State University, Johnson City, TN, United States; ^2^Department of Biomedical Science, Quillen College of Medicine, East Tennessee State University, Johnson City, TN, United States

**Keywords:** every other day fast, spinal cord injury, renal sympathetic nerve activity, cardiovascular regulation, baroreflex

## Abstract

Modest recovery of somatic function after incomplete spinal cord injury (SCI) has been widely demonstrated. Recently we have shown that spontaneous recovery of baroreflex regulation of sympathetic activity also occurs in rats. Dietary restriction in the form of every other day fasting (EODF) has been shown to have beneficial effects on the recovery of motor function after SCI in rats. The goal of this study was to determine if EODF augments the improvement of baroreflex regulation of sympathetic activity after chronic left thoracic (T_8_) surgical spinal hemisection. To determine this, we performed baroreflex tests on ad-lib fed or EODF rats 1 week or 7 weeks after left T_8_ spinal hemisection. One week after T_8_ left hemisection baroreflex testing revealed that gain of baroreflex responsiveness, as well as the ability to increase renal sympathetic nerve activity (RSNA) at low arterial pressure, was significantly impaired in the ad-lib fed but not the EODF rats compared with sham lesioned control rats. However, baroreflex tests performed 7 weeks after T_8_ left hemisection revealed the inability of both ad-lib and EODF rats to decrease RSNA at elevated arterial pressures. While there is evidence to suggest that EODF has beneficial effects on the recovery of motor function in rats, EODF did not significantly improve the recovery of baroreflex regulation of sympathetic activity.

## Introduction

Moment-to-moment regulation of blood pressure is controlled by baroreceptor regulation of sympathetic and parasympathetic activity (Heymans and Neil, 1958; Dampney et al., 2002). Barosensitive neurons are located in the rostral ventrolateral medulla (RVLM) and these neurons mediate the excitability of sympathetic preganglionic neurons located in the intermediolateral cell column of the thoracic and upper lumbar spinal cord (Heymans and Neil, 1958; Dampney et al., 2002). In addition to descending spinal pathways that conduct baroreceptor-mediated excitation of sympathetic preganglionic neurons, there are also spinal pathways responsible for the tonic inhibition of spinally-generated activation of sympathetic preganglionic neurons (Rabchevsky, 2006; Partida et al., 2016). Spinal cord injury (SCI) can seriously impair the sympathetic control of blood pressure. We have previously described the excitatory barosensitive spinal pathways in the rat as bilateral and diffusely located throughout the dorsal and ventral spinal cord, whereas the inhibitory pathways descend the dorsal hemisphere and are ipsilateral (Zahner and Schramm, 2011).

After SCI, orthostatic intolerance results from the inability to produce sympathetically-mediated vasoconstriction when assuming an upright posture, thereby rendering patients susceptible to syncope. Conversely, autonomic dysreflexia occurs due to impaired descending spinal pathways from the brain and brain stem that inhibit sympathetic responses elicited by visceral stimulation caudal to the spinal lesion and results in severe hypertension (Osborn et al., 1990; Krassioukov and Claydon, 2006; Weaver et al., 2006, 2012; Hou et al., 2013). For example, while any stimulus caudal to the lesion may elicit autonomic dysreflexia, it is typically signals from the bladder or bowel that stimulates sensory afferents and also causes sympathetic vasoconstriction (Krogh et al., 1997; Giannantoni et al., 1998). In the intact spinal cord, the sympathetic stimulus for vasoconstriction is inhibited by spinal pathways from the brain and brain stem. However, many times those descending inhibitory pathways are interrupted after thoracic spinal cord injury. The sympathetically induced increase in blood pressure stimulates baroreceptors which signal vasodilation via vasomotor centers in the brain and brain stem (Gao et al., 2002). However, because the sympathetic pathways caudal to the site of injury, as well as the absence of tonically active descending inhibitory pathways are interrupted, blood flow is shunted to vasodilated areas above the site of injury. This inappropriate shunting of blood from the lower extremities results in flushing, sweating, pounding headache, and in some cases seizure, and stroke. Both orthostatic hypotension and autonomic dysreflexia result from interruption of descending sympathetic spinal pathways and impair regulation of spinal sympathetic neurons caudal to the lesion (Osborn et al., 1990; Mayorov et al., 2001; Weaver et al., 2001, 2006; Gao et al., 2002; Krassioukov and Claydon, 2006).

Studies from several laboratories including our own have documented the partial recovery of function after SCI in rats and mice (Weidner et al., 2001; Bareyre et al., 2004; Gulino et al., 2007; Courtine et al., 2008; Mountney et al., 2010, 2013; Beaumont et al., 2014). We have previously shown that baroreflex regulation in spinally-lesioned rats improves over time and that the degree of recovery is dependent on the rostrocaudal location of the lesion (Zahner et al., 2011). Also, using the transsynaptic tracer pseudorabies virus we have shown that partial improvement is likely due to increased efficiency of existing spinal pathways, as opposed to the formation of new spinal pathways to circumvent the lesion site (Castillo et al., 2012).

Negative energy balance in the form of every other day fasting (EODF) has been shown to improve motor and sensory function in rats (Plunet et al., 2008, 2010; Jeong et al., 2011). Additionally, improvements in neural and cognitive function after injury to the central nervous system have also been observed with EODF treatment (Rubovitch et al., 2019). Thus, the goal of this study was to determine if EODF improves baroreflex regulation of sympathetic activity after spinal lesion in the rat. To do this we performed left hemisection at T_8_ of the thoracic spinal cord in 4 separate groups of rats that were fed ad-lib or every other day for 1 week or 7 weeks after the spinal lesion. In EODF rats tested 1 week after the lesion we observed what appeared to be an improvement in baroreflex regulation compared with rats fed ad-lib. However, this apparent improvement did not reach statistical significance. In EODF-treated rats tested 7 weeks after the spinal lesion baroreflex function was not significantly different from that of the ad-lib fed rats and remained significantly impaired compared with the sham-lesion controls. Thus, whereas EODF may improve motor and sensory function there is no sustained improvement in sympathetic cardiovascular control.

## Methods

Female Sprague-Dawley rats (Charles River, Raleigh, NC) weighing between 100 and 250 g were surgically prepared in accordance with the Guide for the Care and Use of Laboratory Animals (National Research Council (US), 1996) using procedures approved by the Johns Hopkins University Committee on Animal Care and Use. Surgeries to perform left spinal hemisection (or sham lesion) were performed in a total of 60 rats. Of these 60 rats, 34 rats underwent surgery for left spinal hemisection and 26 underwent sham surgery. We specifically used female rats because they have a slower growth curve compared with male rats. We performed spinal lesion on rats at an appropriate age such that baroreflex tests would be performed, after a 1-week or 7-week recovery period, on early adult (∼12-week-old) rats. This age of rat correlates to a 24-year-old human (Sengupta, 2013) and was based on our previous studies in the rat (Pan et al., 2005; Mountney et al., 2010, 2013; Zahner et al., 2011; Castillo et al., 2012). 14 rats died due to spinal cord surgery or during the acute renal sympathetic nerve recordings.

### Food and Body Weight

Body weights were recorded before surgery and once every week thereafter. Food and water intake were measured daily. Pre-weighed food and water were left in individually housed rats’ food hoppers and water bottles, respectively, at 5 PM daily. Leftover food and water were weighed 24hr later and the difference recorded.

### Experimental Design

Prior to the spinal lesion, rats were randomized into either ad-lib or EODF for either the 1 week or 7-week protocol. The control groups (ad-lib) had continuous access to food whereas the experimental groups (EODF) were either fasted or had access to chow in 24 h alternating periods. The alternating EODF feeding schedule (or ad-lib control) began immediately after spinal cord surgery and was carried out for either 1 week or 7 weeks post-lesion, depending on the group. All rats had unrestricted access to water. Because baroreflex responses in sham-lesioned were identical in the ad-lib and EODF rats those data were combined into sham-lesion for the respective 1-week or 7-week group (see Supplementary Figures 1, 2).

### Thoracic Spinal Cord Hemisections

For left spinal hemisections rats were anesthetized in a plastic chamber and then with a nose cone, using 2% isoflurane in O_2_. The dorsal surface of the rat was shaved and cleaned with betadine before surgery. A 2 cm cutaneous incision was made centered on the T_6_ and T_7_ vertebra which overly the T_8_ spinal level and a small retractor was used to maintain a clear surgical field. The paraspinal muscles from the dorsal surface of the exposed vertebrae were removed using a septal elevator. After the dorsal surfaces of the vertebrae were free of muscle and connective tissue, the dorsal portion of the vertebra was removed using a micro-rongeur to access the underlying spinal segment. The dura was opened with dura scissors and the left hemisphere of the spinal cord was cut with a 1 mm sapphire blade micro-knife (World Precision Instruments, Sarasota, FL). The dorsal musculature and skin were closed with sterile sutures and wound clips. For sham-lesions, we used an identical technique, but without opening the dura. All rats subjected to survival surgery were injected intramuscularly with the NSAID Banamine (flunixin meglumine, 1.1 mg/kg) before anesthesia was discontinued. During daily post-surgical inspections, rats’ bladders were manually expressed when necessary.

At the end of experiments, rats were euthanized with intravenous injection of α-chloralose (500 mg/kg) followed immediately by transcardial perfusion with buffered saline (pH 7.4), followed by 4% buffered paraformaldehyde (pH 7.4). The spinal cords were removed and post-fixed in 4% paraformaldehyde overnight. Serial horizontal sections of the spinal cord from T1 to T12 were cut with a freezing microtome (40 μm). After processing, sections were mounted on slides and examined microscopically to verify that the lesion completely transected the left hemisphere of the spinal cord while leaving the right hemisphere intact as previously reported (Zahner et al., 2011; Castillo et al., 2012).

### Acute Surgical Preparation

Acute recordings in EODF rats were performed after an ad-lib cycle and no rats were fasted 24hr prior to surgery. For acute baroreflex experiments, rats were initially anesthetized with isoflurane and prepared as previously described (Mountney et al., 2010; Zahner and Schramm, 2011; Zahner et al., 2011; Castillo et al., 2012). Adequate depth of anesthesia was confirmed by the absence of a withdrawal response to a noxious stimulus (tail pinch). The inguinal area of the rat was cleaned and shaved of fur and the right femoral artery was cannulated with PE-50 tubing (Becton Dickinson, Sparks MD) for the measurement of arterial pressure with a PT300 pressure transducer (Grass Instruments, Quincy, MA) connected to a P122 strain gage amplifier (Grass Instruments, Quincy, MA). The ventral surface of the neck was cleaned and shaved of fur and a 2–3 cm midline incision was made and the sternomastoid and sternohyoid were retracted laterally. The left jugular vein was cannulated with PE-50 tubing (Becton Dickinson, Sparks MD) for the administration of α-chloralose anesthesia. This cannula was also used to administer gallamine triethiodide (see below). The trachea was cannulated with a 14G endotracheal tube for mechanical ventilation using a rodent ventilator (CWE, Ardmore, PA). The left and the right femoral vein was cannulated for the separate administration of vasopressor drugs (for details see “Baroreflex Measurement”). A laparotomy was performed to identify and retract the left kidney for the dissection of the left renal sympathetic nerve via a left flank incision. The renal nerve was carefully dissected from the renal vasculature and surrounding tissue with the aid of an operating microscope.

Rats were administered an initial bolus of α-chloralose over 10–15 min (100 mg/kg I.V., Sigma) and slowly transitioned from isoflurane after the completion of the acute surgical preparation. The depth of anesthesia was determined by the corneal reflex before and during the recovery from paralysis or by the variability of renal sympathetic nerve activity (RSNA) and arterial pressure when rats were paralyzed. Rats were maintained at a surgical plane by supplemental doses of α-chloralose (25 mg/kg). Rats were then paralyzed with neuromuscular blocking agent gallamine triethiodide (40 mg/kg I.V., Sigma). Supplemental doses (10 mg/kg) were delivered after the initial bolus injection wore off (∼90 min). Arterial pressure and RSNA were simultaneously recorded with Cambridge Electronic Design Micro1401 hardware and Spike 2^®^ software. Body temperature was monitored with a rectal probe (Physitemp, RET-2 ISO, Clifton, NJ) and it was maintained between 36 and 37°C with a heating lamp.

### Renal Sympathetic Nerve Recording

For RSNA recording the peritoneum was immersed in mineral oil and the nerve mounted on a bipolar hook electrode. The nerve signal was amplified (X 10,000) and bandpass filtered (300–3000 Hz) by an alternating current amplifier (model P511, Grass Instruments). Sympathetic nerve activity was then monitored through an audio amplifier. The sympathetic activity was further processed by rectification and low pass filtering at a time constant of 1 s and both the unprocessed and the rectified/filtered activity with the arterial pressure were recorded. To avoid recording afferent activity the distal end of the renal nerve was cut.

### Baroreflex Measurement

Stable arterial pressure and RSNA were recorded for at least 15 min before baroreflex testing. Basal blood pressure and heart rate were recorded for the 60 s before baroreflex testing. Baroreflex function curves were created by plotting the reflex change in RSNA to increases and decreases in arterial pressure caused by the vasodilator sodium nitroprusside (SNP, 50 μg/ml) and the α-adrenergic agonist phenylephrine (PE, 125 μg/ml) in successive ramped infusions via the left and right femoral vein catheters. First, SNP was administered beginning at a rate of 2.5 ml/h and increased by 2.5 ml/h every 10 s until an arterial pressure of 60 mmHg below baseline or a maximum rate of 25 ml/h was reached. Following SNP administration, rats were allowed sufficient time for arterial pressure and RSNA to recover to baseline levels (∼1–2 min). Once the baseline was met, PE was administered beginning at a rate of 2.5 ml/h and increasing the rate by 2.5 ml/h every 10 s. These infusions produced an approximately linear decrease (SNP) or increase (PE) in arterial pressure at a rate of ∼1.5 mmHg/s. To produce baroreflex function curves the RSNA from baseline to the SNP-induced nadir (60 mmHg below baseline) and the RSNA from baseline to PE-induced peak arterial pressure (60 mmHg above baseline) were analyzed and plotted. Baroreflex function curves were fit to a sigmoidal function (McDowall and Dampney, 2006). We used the maximum slope of the sigmoidal curves as our measurement of the gain of the baroreflex.

### Data Analysis

Each RSNA response to changes in arterial pressure was fit to sigmoidal function using Prism^®^ software (Graphpad, version 4.0). The sigmoidal function is described by the following equation:

y=A⁢1/(1+e⁢x⁢p⁢(A⁢2⁢(x-A⁢3)))+A⁢4

where y is the RSNA, x is arterial pressure, A_1_ is the range of RSNA, A_2_ is the gain coefficient, A_3_ is the value of x at the midpoint, and A_4_ is the minimum RSNA of the reflex curve (McDowall and Dampney, 2006). For all rats, this sigmoidal function was best fit to the RSNA-arterial pressure relationship.

To construct grouped baroreflex relationship curves, arterial pressure, and corresponding RSNA data were averaged into 10 mmHg bins. To account for variations in baseline arterial pressure we used the change from baseline as the reference for the arterial pressure bins when constructing the baroreflex relationship curves. For the baroreflex relationship curves, the data were expressed as mean change in arterial pressure (in mmHg) from baseline and the % change in RSNA. Stable plateau values were determined when RSNA for a particular 10 mmHg arterial pressure bin did not vary by more than 5% from the previous RSNA value in that 10 mmHg arterial pressure bin. The maximum RSNA plateau occurred typically at least 50 mmHg below baseline arterial pressure, and the minimum RSNA plateau at least 30 mmHg above baseline arterial pressure. The grouped data are expressed as means ± SE. Statistical analyses employed two-way ANOVA (with Holms-Sidak post-tests, see figure legends). Values of *P* < 0.05 were considered significant.

## Results

### Food, Water, and Body Weight

**FIGURE 1:**
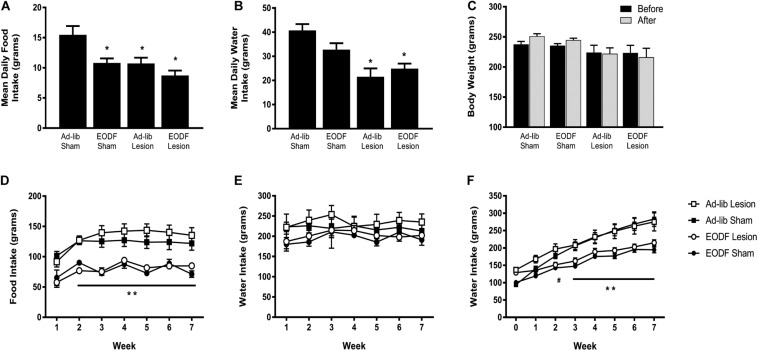
Grouped data showing the mean daily food intake **(A)**, water intake **(B)**, and weekly body weight **(C)** in rats 1 week after sham-lesion or T_8_ spinal hemisection and fed daily (Ad-lib sham *n* = 4, Ad-Lib lesion *n* = 5) or every other day (EODF, sham *n* = 5, EODF lesion *n* = 6). Grouped data showing the mean weekly food intake **(D)**, water intake **(E)**, and weekly body weight **(F)** in rats with sham-lesion or T_8_ spinal hemisection and fed either daily (ad-lib sham *n* = 7, ad-lib lesion *n* = 5) or every other day (EODF, sham *n* = 5, EODF lesion *n* = 9). Data are represented as means ± SE (*significantly different from ad-lib sham, **EODF lesion and EODF sham significantly different from ad-lib lesion and ad-lib sham, ^#^EODF sham and EODF lesion significantly different from ad-lib sham, *P* < 0.05). Two-way ANOVA with lesion and diet as factors was used to analyze **(A,B)** and two-way repeated-measures ANOVA with lesion, diet, and time (before and after or week 0–7) as factors **(C–F)** with Holms-Sidak post-test.

Daily food and water intake, and weekly body weight were measured in groups of rats with T_8_ left hemisection or sham-lesion, and fed every other day ad-lib for 1 week or 7 weeks after spinal lesion. Two-way ANOVA of food intake data in rats given a 1-week recovery period after lesion (or sham) and feed ad-lib or EODF revealed a main effect of diet [*F*(1, 16) = 12.21, *p* = 0.0030] and lesion [*F*(1, 16) = 11.58, *p* = 0.0036] and no significant interaction. *Post hoc* analysis in these groups of rats given a 1-week recovery period after spinal lesion showed that the ad-lib sham group of rats ate significantly more than all other groups (*P* < 0.05, Figure 1A). Two-way ANOVA of water intake data revealed a main effect of diet [*F*(1, 16) = 21.02, *p* = 0.0003]. *Post hoc* analysis showed that water intake in the ad-lib and EODF lesion groups of rats was significantly reduced compared with ad-lib sham groups of rats (*P* < 0.05, Figure 1B). In rats tested 1-week after surgery, there was no significant effect of lesion or diet on body weight (Figure 1C).

In the groups of rats given a 7-week recovery period after lesion (or sham) and feed ad-lib or EODF, two-way ANOVAs of food intake data for diet or lesion and time after recovery revealed a main effect of diet Ad-lib vs. EODF, [*F*(1, 24) = 51.90, *p* < 0.0001] and time [*F*(6,144) = 15.98, *p* < 0.0001] with no significant interaction, and a main effect of time when comparing sham vs. T_8_ hemisection [*F*(6, 144) = 15.50, *p* < 0.0001] with significant interaction [*F*(6, 144) = 2.191, *p* = 0.0471]. *Post hoc* analysis showed that rats subjected to 7 weeks of EODF had significantly lower weekly food intake compared with that of the ad-lib groups of rats after the first week which persisted throughout the study (*P* < 0.05, Figure 1D). Water intake in the 7-week spinal lesion rats did not differ between the ad-lib and EODF groups of rats (Figure 1E). Two-way ANOVA of body weight data for diet (EODF vs. Ad-lib) revealed a main effect of diet [*F*(1, 24) = 17.68, *p* = 0.0003] and time [*F*(7, 168) = 192.9, *p* < 0.0001] with a significant interaction [*F*(7, 168) = 15.96, *p* < 0.0001]. Two-way ANOVA of body weight data for lesion (sham vs. T_8_ hemisection) and time revealed a main effect for time [*F*(7, 168) = 134.3, *p* < 0.0001] with significant interaction [*F*(7, 168) = 4.304, *p* = 0.0002]. *Post hoc* analysis showed that the mean body weight of 7-week EODF rats was significantly less than that of the ad-lib rats from week 3 and thereafter (*P* < 0.05, Figure 1F).

### Baroreflex Function

**FIGURE 2:**
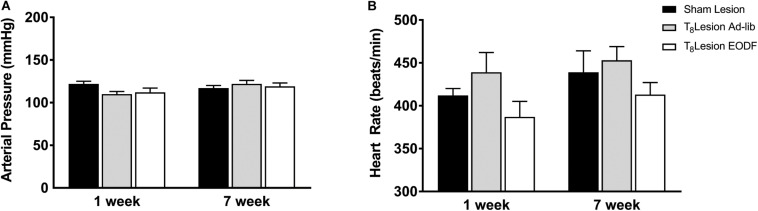
Group data showing the arterial pressure **(A)** and heart rate **(B)** 1 week or 7 weeks sham lesion (1-week *n* = 9, 7-week *n* = 12) or left hemisection and fed either ad-lib daily (1-week *n* = 5, 7-week *n* = 5) or every other day (EODF; 1-week *n* = 6, 7-week *n* = 9). Data are represented as means ± SE. Data were analyzed using two-way ANOVAs with group (sham lesions, ad-lib lesion, EDOF lesion) and time (1, 7 weeks) as factors with Holms-Sidak post-test.

**FIGURE 3:**
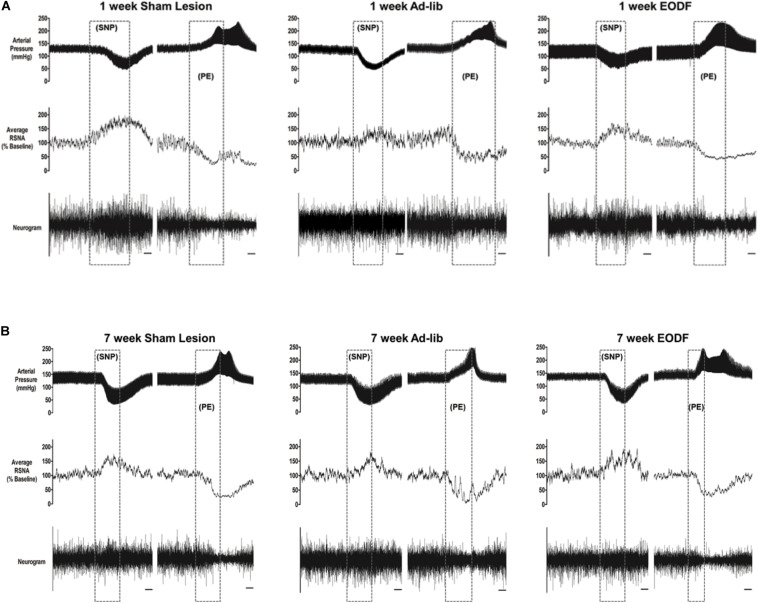
Representative tracings from rats in which baroreflex responsiveness of renal sympathetic nerve activity (RNSA) was performed 1 week **(A)** or 7 weeks **(B)** after either sham lesion or T_8_ left hemisection and fed either ad-lib daily or every other day (EODF). Tracings show the arterial pressure, left RSNA, and the baroreflex response in RSNA to changes in arterial pressure. Changes in arterial pressure were induced by I.V. infusion of sodium nitroprusside (SNP) and phenylephrine (PE). Scale bar equals 10 s.

To determine if EODF augments the spontaneous recovery of sympathetic cardiovascular regulation after T_8_ left-hemisection we measured blood pressure, heart rate, and RSNA and performed baroreflex tests. Prior to baroreflex testing the basal mean arterial pressure and heart rate in both ad-lib and EODF rats with T_8_ hemisection measured 1 week and 7 weeks after spinal hemisection was not significantly different compared with that of the sham-lesioned rats (Figure 2). Representative tracings of basal arterial pressure, RSNA, and baroreflex responses of RSNA in a sham-lesioned and a T_8_ left-hemisected rat fed either ad-lib or every other day for 1 week or 7 weeks after surgery are shown in Figure 3.

**FIGURE 4:**
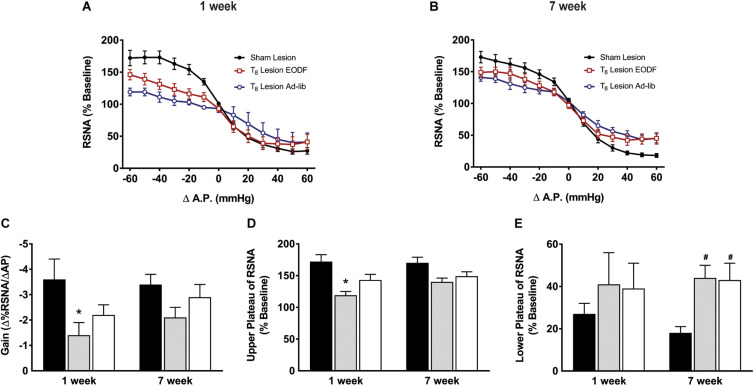
Grouped data showing baroreflex relationship of arterial pressure and left RSNA from sham-lesioned (*n* = 12), ad-lib (*n* = 5), and EODF (*n* = 9) rats 1 week **(A)** or 7 weeks **(B)** after T_8_ left hemisection. Maximal gain of the baroreflex response **(C)**, maximum plateau of RSNA during baroreflex testing **(D)**, and minimum plateau of RSNA during baroreflex testing **(E)**. Data are represented as means ± SE. Data were analyzed using two-way ANOVAs with group (sham lesions, EDOF + lesion, lesion) and time (1 week, 7 weeks) as factors with Holms-Sidak post-test. ^∗^Significantly different from 1-week sham, ^#^significantly different from 7-week sham, *P* < 0.05.

Grouped baroreflex curves are shown in Figures 4A,B. During analysis of baroreflex data two-way ANOVA showed a main effect of lesion on gain [*F*(2, 40) = 4.301, *p* = 0.0203] upper plateau [*F*(2, 40) = 8.964, *p* = 0.0006], and lower plateau [*F*(2, 40) = 3.985, *p* = 0.0264]. *Post hoc* analysis showed that the mean maximum gain of the baroreflex in the T_8_ left-hemisectioned rats given 1-week of recovery and fed ad-lib was significantly impaired compared with the sham-lesioned rats (*P* < 0.05, Figure 4C). The baroreflex-induced increase in RSNA was significantly impaired in T_8_ left-hemisectioned rats fed ad-lib (*P* < 0.05) but not in the EODF T_8_ left-hemisectioned rats compared with the sham-lesioned rats (Figure 4D). The mean baroreflex-induced decrease in RSNA was not significantly impaired in T_8_ left-hemisectioned rats fed ad-lib or EODF compared with the sham-lesioned rats. In rats tested 7 weeks after T_8_ left-hemisection neither the mean maximum gain of the baroreflex nor the baroreflex-induced increase in RSNA was significantly different in the ad-lib or EODF rats compared with that of the sham-lesioned rats. However, the baroreflex-induced decrease in RSNA was significantly (*P* < 0.05, Figure 4E) impaired in both the T_8_ left-hemisectioned rats fed ad-lib and EODF compared with that of the sham-lesioned rats.

## Discussion

Caloric restriction has been reported to be neuroprotective and to increase functional recovery after SCI in the rat (Plunet et al., 2008, 2010; Jeong et al., 2011). While EODF has been shown to augment the recovery of motor and sensory function after SCI (Plunet et al., 2008, 2010; Jeong et al., 2011), data on the extent of recovery of sympathetic cardiovascular regulation are limited. Based on studies from our laboratory to show recovery of baroreflex control of sympathetic activity after chronic SCI (Zahner and Schramm, 2011; Zahner et al., 2011; Castillo et al., 2012), as well as studies to suggest that caloric restriction augments baroreflex responsiveness in the naïve rat with intact spinal cords (Herlihy et al., 1992; Thomas et al., 1993), it was our intent to determine if EODF also augments the recovery of baroreflex regulation of sympathetic activity after SCI. Even though others have shown that EODF augments the recovery of motor and sensory function, this study provides important new physiological evidence to show that chronic EODF does not improve the impaired baroreflex regulation ability to suppress sympathetic activity at elevated blood pressures in rats after SCI.

Improvements in sensory and motor function after SCI are well documented in both the rat and mouse (Weidner et al., 2001; Bareyre et al., 2004; Ballermann and Fouad, 2006; Gulino et al., 2007; Courtine et al., 2008; Plunet et al., 2008, 2010; Mountney et al., 2010, 2013; Jeong et al., 2011). Some recovery in these models can be attributed to robust neuronal sprouting and corticospinal tract collateralizations after injury (Fouad et al., 2001; Hill et al., 2001; Weidner et al., 2001; Bareyre et al., 2004; Pan et al., 2007). Restoration of function is also attributed to the reorganization of new and spared propriospinal connections that contribute to the “re-routing” of pathways (Stelzner and Cullen, 1991; Bareyre et al., 2004; Courtine et al., 2008). To assess recovery of spinal pathways involved in baroreflex sympathetic regulation in this study and others we measured left RSNA after a T_8_ left hemisection and therefore refer to the left spinal pathways as “ipsilateral” (Zahner and Schramm, 2011; Zahner et al., 2011; Castillo et al., 2012). In previous studies, we have shown that in the intact rat, the pathways responsible for baroreflex-induced increases in RSNA at reduced arterial pressure descend bilaterally and the pathways responsible for baroreflex-independent, tonic inhibition of ongoing spinally-generated sympathetic activity descends mainly ipsilaterally (Zahner and Schramm, 2011). In that study, we showed that immediately following acute ipsilateral T_8_ left hemisection, rats retained some ability to increase left RSNA upon decreases in arterial pressure due to uninterrupted contralateral pathways. However, the ability to decrease RSNA at elevated arterial pressure is significantly attenuated, which is likely due to reduced descending tonic inhibition of ongoing, spinally-generated RSNA which is mainly ipsilateral. In another study, we showed in the chronic rat hemisection model, that baroreflex control of ipsilateral RSNA improves, to some degree, over time following the initial lesion (Zahner et al., 2011). This modest recovery of baroreflex control of sympathetic activity is not due to “new” pathways that circumvent the lesion but rather it is likely due to increased efficacy of already existing pathways that were not directly affected by the surgical spinal hemisection (Castillo et al., 2012). In the current study, we also observed an attenuated ability to reduce left RSNA (ipsilateral to the lesion) at elevated blood pressure in rats 7 weeks after a left T_8_ hemisection. We did observe modest improvements in the ability to increase left RSNA during low blood pressure, however this improvement was not augmented with EODF. Although histological evidence is lacking, we can speculate that regardless of EODF treatment the spontaneous improvement of baroreflex function is better in the bilateral descending excitatory pathways than in the ipsilateral descending inhibitory pathways.

An important difference between the current study and those that examined the effect of EODF in the recovery of locomotor activity after SCI is that in those studies SCI was induced with contusion injury (Plunet et al., 2008, 2010; Jeong et al., 2011) as opposed to surgical hemisection in the current study. While we have also shown recovery of motor function using contusion lesion in the rat we have found the recovery of sympathetic baroreflex regulation after contusion lesion to be quite variable (Mountney et al., 2010, 2013). In a study by Saruhashi et al. (1996) using the surgical spinal hemisection model they found that ipsilateral but not contralateral 5-HT is diminished after lesion and that the return of ipsilateral spinal 5-HT immunoreactivity is positively correlated with the recovery of locomotor function (Saruhashi et al., 1996). As such, one possible mechanism in the spontaneous recovery of spinal sympathetic activity may also be the return of 5-HT expressing neurites as well. In this regard, increased spinal 5-HT may also correlate to improved baroreflex function. However, because the improvements in the baroreflex response are more subtle compared with the recovery of locomotor activity, EODF alone may not be sufficient stimulus to augment the recovery of sympathetic baroreflex activity after spinal cord injury.

While it appeared that 1 week after spinal hemisection that EODF may attenuate the impairment of both gain and the ability to increase sympathetic activity at low blood pressure (upper plateau), in rats tested 7 weeks after lesion neither of these measurements were significantly different from each other or sham-lesioned control group. Consequently, EODF does not appear to enhance the recovery more than that which occurs spontaneously. While EODF treatment did not improve baroreflex activity after spinal hemisection the lack of improvement may not be specifically due to a lack of neural plasticity. Although it is likely that neural plasticity after spinal hemisection contributes to the spontaneous recovery of baroreflex function, we must not discount non-neural components, such as changes in arterial stiffness following SCI and that may play an important component in the recovery of baroreflex activity (Phillips et al., 2012, 2014). Although we did not consider the sexual maturity or cycle stage of these female rats in this study, the role of sex hormones in the recovery of function is an important consideration. A study by Farooque et al. (2006) shows that the extent of spinal cord tissue injury after impact lesion is less severe and the recovery of motor function is significantly better in adult female vs. male rats (Farooque et al., 2006). In another study, 17β-estradiol treatment in male rats, also reduced the size of the lesion and augmented motor recovery (Yune et al., 2004). However, a more recent study by Walker et al. (2019) in which they performed a histological and functional comparison of gender in age-matched rats showed that moderate thoracic contusion injury is not different between sexes (Walker et al., 2019). Nevertheless, sex as a biological variable in spinal cord injury and the role of sex-hormone related treatments as well as studies to better characterize the spinal mechanisms in the spontaneous recovery of baroreflex activity are needed.

### Clinical Significance

Because the locations of spinal cord lesions have a direct consequence on the severity of orthostatic hypotension and/or autonomic dysreflexia after SCI (Furlan et al., 2003), it is very important to develop treatments that can augment the plasticity of spinal sympathetic pathways. In the future, it will be important to determine the spinal mechanism of recovery. It is also important to determine whether treatments that have already shown to improve somatomotor and somatosensory function can also improve the recovery of sympathetic cardiovascular function. While the results from this study may at first appear discouraging they are nevertheless important steps toward better understanding and improving the recovery of sympathetic cardiovascular function following spinal injury.

## Data Availability Statement

The datasets generated for this study are available on request to the corresponding author.

## Ethics Statement

The animal study was reviewed and approved by the Johns Hopkins University.

## Author Contributions

MZ provided study concept, design, acquisition of data, and analysis and interpretation of data. MZ and EB drafted the manuscript. EB provided important intellectual advice on the data and valuable editorial input to the manuscript. Both authors contributed to the article and approved the submitted version.

## Conflict of Interest

The authors declare that the research was conducted in the absence of any commercial or financial relationships that could be construed as a potential conflict of interest.
