# Supplementary figures and images for: Intermittent Fasting After Spinal Cord Injury Does Not Improve the Recovery of Baroreflex Regulation in the Rat
Source: Front Physiol. 2020 Jul 22;11:865. doi: 10.3389/fphys.2020.00865 (PMC7387690; doi:10.3389/fphys.2020.00865)

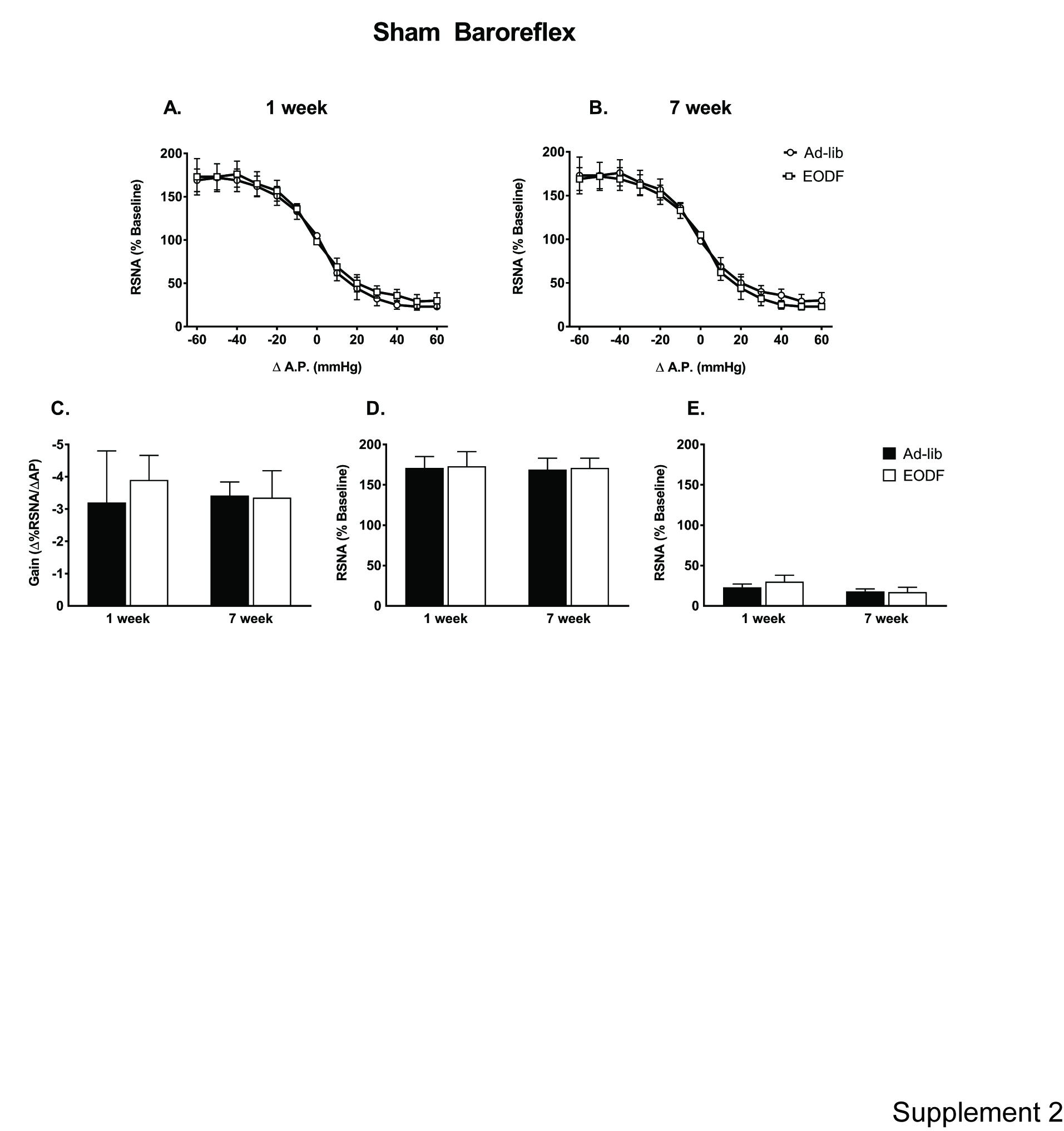

Supplement: Supplementary file 1 [file Image_1.TIF]

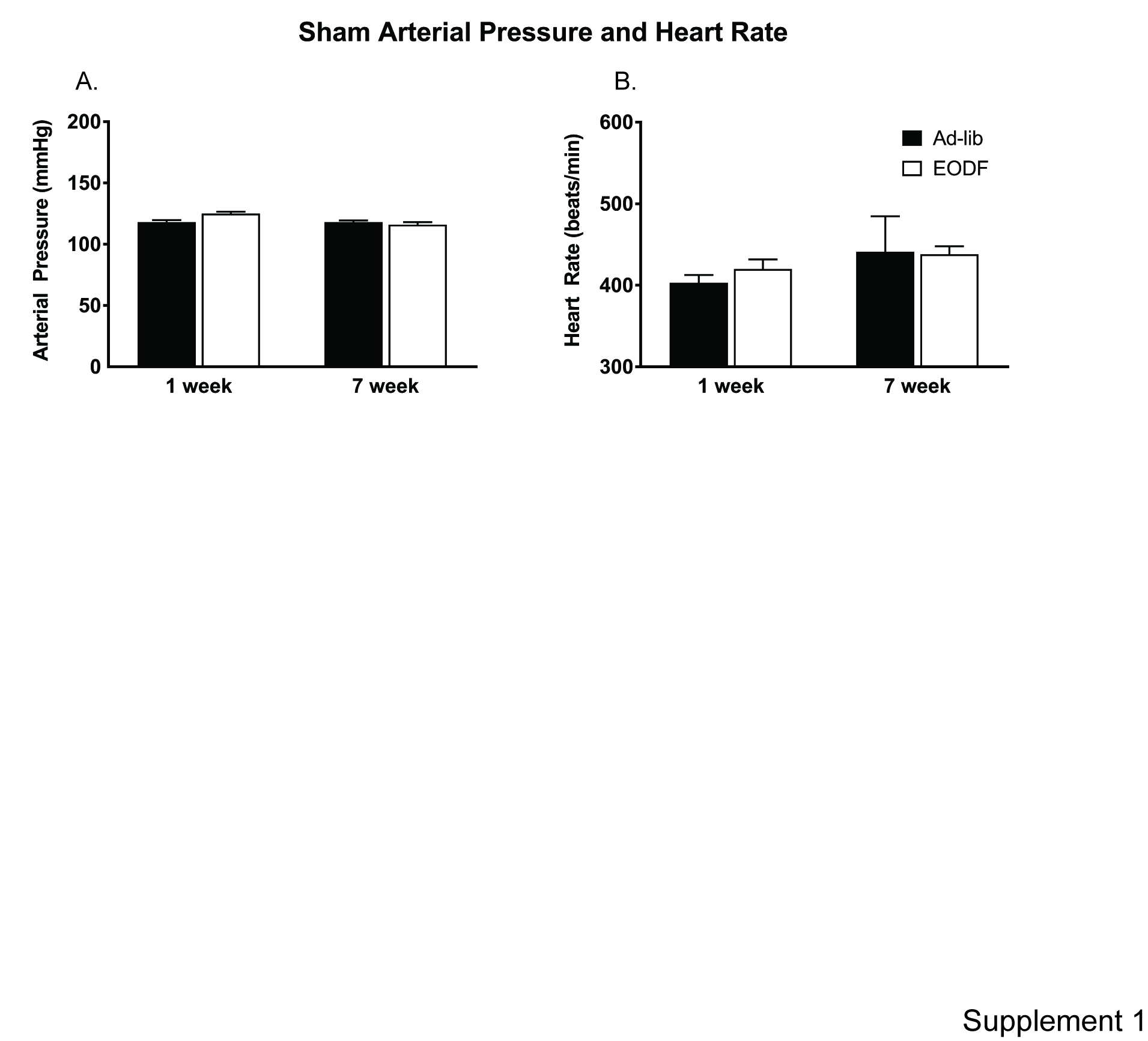

Supplement: Supplementary file 2 [file Image_2.TIF]
