# Supplementary material for: Intermittent Fasting After Spinal Cord Injury Does Not Improve the Recovery of Baroreflex Regulation in the Rat
Source: Front Physiol. 2020 Jul 22;11:865. doi: 10.3389/fphys.2020.00865 (PMC7387690; doi:10.3389/fphys.2020.00865)
Supplement: Supplementary file 3 [file Data_Sheet_1.docx]

# Supplemental Figure Legends

Supplemental Figure 1. Grouped data showing no difference in basal blood pressure (A) or heart rate (B) 1 week or 7 weeks after sham lesion and fed either ad-lib daily (1-week n = 4, 7-week n = 7) or every other day (EODF; 1-week n = 5, 7-week n = 5). Data are represented as means ±SE and analyzed using two-way ANOVAs with group (sham lesions, ad-lib lesion, EDOF lesion,) and time (1 week, 7 weeks).

Supplemental Figure 2. Grouped data showing no difference in the baroreflex curves in rats fed ad-lib or every other day (EODF) and tested 1 week (A) or 7 weeks (B) after sham lesion and fed either ad-lib daily (1-week n = 4, 7-week n = 7) or every other day (EODF; 1-week n = 5, 7-week n = 5) maximal gain of the baroreflex response (C), maximum plateau of RSNA during baroreflex testing (D), and minimum plateau of RSNA during baroreflex testing (E). Data are represented as means ±SE and analyzed using two-way ANOVAs with group (sham lesions, ad-lib lesion, EDOF lesion,) and time (1 week, 7 weeks).
